# Supplementary material for: Are carbon emissions trading and green financial instruments synergistic? -Comprehensive quantitative research based on content analysis
Source: PLoS One. 2024 Mar 7;19(3):e0298601. doi: 10.1371/journal.pone.0298601 (PMC10919601; doi:10.1371/journal.pone.0298601)
Supplement: S1 Data — (ZIP) [file pone.0298601.s001.zip › Data/地级市Z_F工作报告.zip!/╜¡╦╒/╨∞╓▌╩╨/└┤╫╘╣1⁄2╓┌║┼í╛┬φ┐╦╩2╛▌═°í┐_2014.docx]

各位代表：

现在，我代表市人民政府向大会作工作报告，请予审议，并请市政协各位委员和其他列席人员提出意见。

一、2013年工作回顾

过去一年，是全面贯彻落实党的十八大精神的开局之年，也是宏观形势更加错综复杂、经济下行压力加大、各种困难明显增多的一年。面对严峻挑战，在省委、省政府和市委的坚强领导下，全市上下始终紧扣主题主线，深入实施“八项工程”，全力突破“三重一大”，一着不让稳增长、调结构、惠民生、促和谐，各项工作在攻坚克难中取得了新成效。

（一）经济发展稳中有进，转型升级步伐加快

经济保持平稳增长。全市主要经济指标增长继续高于全省平均水平，完成（预计，下同）地区生产总值4500亿元、增长12%左右；实现公共财政预算收入422.8亿元、增长15.3%，其中税收收入增长20.1%、占比较上年提高3.2个百分点；完成固定资产投资3131亿元、增长22%，其中工业投资1660亿元，服务业投资增长29%、占比提高3.1个百分点；实现社会消费品零售总额1496亿元、增长14%。亿元以上项目开工775个、完成投资1800亿元，徐工全地面起重机、布兰肯矿山设备、美驰车桥二期、时代天虹三期等项目建成投产，亿吨大港、黄河故道综合开发、雨润物流中心、必康新医药等项目顺利实施。县域实力加快壮大，“突破睢宁”战略和“丰县崛起”行动计划扎实推进，邳州市和新沂市通过省全面达小康评审认定，沛县通过生态县省级验收。

产业结构不断优化。工业总产值突破万亿元，其中六大千亿元工业产业实现总产值9100亿元；规模以上工业增加值完成2315亿元、增长13%。服务业增加值完成1920亿元，占比提高1个百分点，50个现代服务业集聚区入园企业比上年增加1619家。新增2家省级文化产业示范基地和2家省级生态旅游示范区，淮海文博园跻身省级服务业集聚区，龟山景区成功创建4A级旅游景区，创意68产业园被列入首批省级广告产业园，我市获批全国电子商务示范城市。粮食生产实现十连丰，新建和改造设施农业20万亩，高效设施农业发展继续领跑全省。高新技术和新兴产业产值分别实现3485亿元和3700亿元，占规模以上工业比重分别达到34%和36%。新增超百亿元企业（园区）1家、省两化融合试点（示范）企业38家、省级高新技术企业40家、省级农业龙头企业17家。

改革创新持续深化。县（市）区科学发展综合考核、市直机关单位绩效考核、乡镇分类考核机制进一步完善，对城区、开发区简政放权力度加大，市级扩权强镇改革及经济发达镇行政管理体制改革试点有序实施，部分镇改设街道办事处全面完成。创新农业经营机制，新增农田托管75万亩、适度规模经营52万亩、土地流转55万亩，均居全省第一；家庭农场、农民专业合作社分别发展到1872家和9371家。实施县级医院综合改革，取消药品加成政策；采取政府购买服务的方式拓展基本药物制度覆盖范围，为群众减轻药品负担3.8亿元；实现相邻四省的58个县（市、区）新农合异地结算报销。创新政府投融资体制，规范管理政府性债务，有效整合资源加强投融资平台建设，政府融资平台投融资能力明显提升。“彭城英才”计划扎实推进，新引进高层次创新创业人才400多人。新增专业技术人才3.86万人、校企联盟210家，研发机构实现规模以上工业企业全覆盖。我市连续四次获得“全国科技进步先进市”称号。

开放领域逐步拓宽。成功举办第十六届投资洽谈会、香港合作投资推介会、上海跨国公司联谊会、新加坡江苏合作理事会第七次会议、首届国际工程机械交易会等十余项重大招商活动，一批重大投资项目签约落地。全市实际到帐注册外资15亿美元，进出口总额60亿美元、其中出口44亿美元，服务贸易进出口额7.2亿美元、同比增长77%。对外交流合作进一步深化，观音机场年旅客吞吐量突破100万人次，至韩国首尔、台湾高雄航线如期开通；与新加坡合作共建的伊顿国际学校落户新城区。民营经济实现增加值2650亿元、增长13%，注册资本2455亿元、增长16.8%，年内新增民营企业和个体经营户5.4万个。

（二）城镇化进程不断加快，城乡面貌加速提升

中心城市功能进一步增强。围绕“八大中心”建设，精心实施城市总体规划修编，八大类255项城建重点工程开竣工数量再创新高。韩山隧道、老字号街区、万达广场、万科一期、残疾人康复中心等项目竣工使用，三环东路高架基本建成，郑徐客专、城市轨道交通、三环西路高架启动实施，丰沛铁路全线贯通，“两河两路”、苏宁商务广场等项目加快推进，中心城市综合功能和集聚辐射力加速提升。“外创示范道路、内创幸福家园”活动深入开展，全市省级示范路达7条，幸福家园示范小区达54个。数字城管系统平台加速构建，网格化管理机制进一步完善，占道经营、户外广告等市容专项整治效果明显。我市成为国土部首批3个“智慧城市”建设试点和规划重点发展的23个都市圈之一。

新型城镇体系初步构建。“1+3个100万”工程有序实施，5县（市）老城改造和新城建设同步推进。中心镇建设步伐加快，30个集中创建镇投资125亿元完成各类项目248个，完成第二批12个创建镇达标验收，66个镇创建管理示范镇工作全面启动。新增新农村示范村50个，省三星级康居乡村达59个；新建农村公路1225条，镇村公交覆盖率达25%以上；完成农村水利投资9.21亿元，疏浚县乡河道1188公里；新增农村有线电视用户11.6万户；新增改厕15万户。

生态建设力度明显加大。“五大行动计划”启动实施，国家生态市和国家生态园林城市创建加速推进。多措并举整治空气污染，强力实施秸秆禁烧、燃煤禁烧、扬尘治理和热电整合，关停城区3家小热电企业，淘汰黄标车11731辆。全市建成污水处理厂87座，污水集中处理率达到88.6%。投资13亿元对82条河流实施河道清淤和生态修复，南水北调东线一期建成通水，6个断面水质基本达标，我市跻身全国首批水生态试点城市。新增造林12万亩，“二次进军荒山”完成造林1.9万亩，全市建成区绿化覆盖率41.8%，公园绿地500米服务半径覆盖率89%，森林覆盖率32%、保持全省领先。完成31家工业企业退城入园，单位地区生产总值能耗下降4%，落后产能淘汰和主要污染物减排完成省定任务。城乡环境综合整治成效明显，京沪高铁沿线环境整治和2179个村庄环境整治工作全面完成。

（三）人民生活持续改善，社会保持和谐稳定

民生实事得到有效落实。三大类100项为民办实事项目年度任务全面完成，保障房建设、医院新建迁建、社区卫生服务中心提档升级、邻里中心建设、市民卡（社保卡）、关爱老人、残疾人帮扶等项目顺利实施，发放自行车卡36.2万张，公共自行车增至1.64万辆，新增500辆公交车。全市城乡居民收入分别为23670元和12050元，增长9%和12%。大力促进就业创业，城镇新增就业11.7万人、失业人员再就业8.64万人、农村劳动力转移6万人，完成就业创业培训11.9万人，城镇登记失业率控制在2.14%；建成大学生创业园18个，大学生创业企业达到3800家，累计提供就业岗位近3万个。强化扶贫开发，发放扶贫小额贷款12.2亿元，11.6万户低收入农户从中受益。完成棚户区和城中村改造1500余万平方米，建成安置房401.6万平方米。

保障体系更加健全完善。社会保险覆盖面进一步扩大，参保人数超过620万人；新农合参合率连续7年稳定在95%以上，住院实际补偿比例达到57.5%；城乡低保标准分别提高到每人每月510元和270元，保持苏北领先；启动城乡居民大病保险和医保“二次补助”，补助额5130万元，有效解决了部分困难群众因病致贫问题。医疗救助、重残救助、临时生活救助体系不断完善，“三无”人员和孤儿供养标准进一步提高。开工建设保障性住房7528套，建成7193套，销售（配租）7796套，发放廉租房补贴1401户。

各项社会事业协调发展。以县为单位全面实现教育基本现代化，6个县（市）区通过“全国义务教育发展基本均衡县（市、区）”国家级验收；新建改扩建幼儿园128所、农村中小学44所，加固重建中小学校舍75万平方米，职业教育创新发展实验区有序推进，教育办学条件和均衡发展水平不断提高。中心医院新城区分院、徐医附院开发区分院等项目加快实施，新增省级示范卫生院13个，改造升级社区卫生服务中心（站）42个，医疗资源布局更趋优化。第十八届省运会筹办工作扎实推进，奥体中心基本建成，全民健身活动广泛开展，顺利建成江苏省体育强市。“舞动汉风”工程深入实施，文艺精品创作成果丰硕。文化惠民活动扎实开展，送戏下乡520场、电影2.89万场、图书11.1万册。人口自然增长率控制在6‰以内，出生人口性别比升高的趋势得到遏制。人防、民兵、双拥、民族宗教、档案、外事、侨务、台湾事务、统计、审计、新闻出版、住房公积金管理、无线电管理、淮塔管理、机关事务管理、应急管理、防震减灾、气象、妇女儿童、青少年、关心下一代、援疆等各项事业都取得了新的进步。

社会管理创新全面加强。深入实施“四治理三提升”工程，“一委一居一站一办”和网格化管理服务模式实现全覆盖，城乡和谐社区达标率分别达到75%和55%以上，刑事案件发案数下降8.2%，我市荣登中国最安全城市排行榜第6位。创新实施专职人民调解员进驻公安派出所的公调对接机制，积极推进“阳光信访”，重大事项稳评率达到100%，一批信访突出问题得到较好解决。抓好安全生产、食品药品和产品质量监管，各类事故和死亡人数分别下降15.7%和15.9%，杜绝了较大以上安全生产事故的发生。

（四）政府自身建设扎实推进，发展环境更趋优化

行政效率效能和服务水平得到提升。认真落实中央八项规定和省委、市委十项规定，“三解三促”活动实现常态化制度化。深入实施百姓办事“零障碍”工程，优化服务流程85个，精简审批事项113个，清除办事障碍850项。“12345”政府服务热线覆盖面进一步扩大、功能向群众生活领域延伸，受理群众投诉17.9万件，办结率97%。创新信息公开形式，集新闻发布、网络发言和政务微博三位一体的“徐州发布”启动运行。全面推行“两参一改”，持续深化行风评议，有力促进了机关作风转变。

推动发展和破解难题的能力得到提升。与金融机构的战略合作进一步深化，全市各类银行业金融机构达到37家；人民币贷款余额比年初增加313.6亿元，非信贷直接融资规模不断扩大。土地开发复垦整理、工矿废弃地盘活利用、城乡建设用地增减挂钩及万顷良田建设深入实施，在保障用地需求的同时，实现了耕地总量动态平衡；土地节约集约利用不断强化，盘活利用存量土地1.2万亩，建成和在建高标准厂房322万平方米。创新财政资金扶持方式，深化银企对接、企业金融顾问制度；扎实推进“营改增”工作，累计减免企业税赋13亿元。

依法行政和廉洁从政水平得到提升。自觉接受市人大及其常委会的法律监督和工作监督、市政协及社会各界的民主监督，加强行政执法规范化建设，力求把依法办事贯穿于政府工作各个环节。厉行勤俭节约，除涉密部门外的市级部门预算全面向社会公开，市直公务接待、公车购置和公车运行、因公出国费用分别下降15.79%、15.82%和26.8%。继续实施反腐倡廉“八项工程”，扎实推进“两个习惯”教育，深入开展领导干部“晒权力”试点和机关处长“晒职权”活动，始终保持惩腐肃贪的高压态势。

各位代表，过去一年各项成绩的取得来之不易，这是全市人民团结拼搏的结果，是社会各界共同努力的结果。在此，我代表市人民政府，向全市各族人民群众，向人民解放军驻徐部队、武警官兵和公安民警，向各民主党派、工商联、无党派人士和人民团体，向支持徐州现代化建设的香港特别行政区同胞、澳门特别行政区同胞、台湾同胞、海外侨胞和各界人士，向投身徐州经济社会发展的国内外投资者和企业家，表示衷心的感谢！

在肯定成绩的同时，我们也清醒地看到：虽然我市主要经济指标增速好于全国全省，但受宏观形势影响，社会消费品零售总额、进出口总额、实际到帐注册外资等指标增速低于预期。内需不旺和外需不振并存，工业产业投入后劲不足，居民消费增速回落，外贸出口出现大幅下降，部分企业受增本减利因素影响生产经营困难。产业结构偏重的问题尚未根本改观，资源环境硬约束加剧，节能减排形势依然严峻，自主创新能力亟待提高。城市交通拥堵、空气污染等问题凸显，县域城镇化水平不高，部分镇村脏乱差的现象仍比较突出。财政减收增支因素增多，居民收入增速趋缓，保障和改善民生的压力加大。政府职能转变需要进一步加快，个别部门存在作风不实、效能不高、监管职能履行不到位的现象。我们将以对人民高度负责的使命感和责任感，继续下大力气解决存在的矛盾和问题，扎实做好政府各项工作，决不辜负全市人民的重托和期望。

二、2014年工作总体要求

今年是贯彻落实党的十八届三中全会精神、全面深化改革的第一年，也是我市以县为单位全面建成小康社会的决胜之年。综观今年的宏观形势，国际和国内、有利和不利、长期和短期、结构性和周期性因素相互交织，发展环境更加复杂。一方面，多种积极因素持续积累。世界经济出现缓慢回升迹象，总体态势趋稳；国家继续实施积极的财政政策和稳健的货币政策，保持宏观经济政策的连续性和稳定性，国内经济运行质量和效益持续改善，特别是各项改革的全面深化、城镇化步伐的不断加快，将为经济社会发展提供强大的内生动力。另一方面，各种风险挑战叠加冲击。全球经济复苏基础仍不牢固，主要经济体增长低位徘徊，国际市场争夺更加激烈，美国收紧量化宽松货币政策需要高度关注，世界经济运行中不稳定不确定因素依然较多；我国经济发展正处于增长速度换挡期、结构调整阵痛期、深化改革深水期、前期政策消化期，外需减弱、部分行业产能相对过剩、企业生产经营成本上升，经济下行压力不可低估。我市发展既面临交通枢纽优势不断强化、省实施新一轮振兴徐州老工业基地扶持政策等机遇，但在结构调整、生态建设、节能减排、民生改善等方面也面临更重的压力和任务。我们既要看到有利条件和积极因素，坚定科学发展信心不动摇，又要充分估计面临的困难和挑战，主动作为、趋利避害，在克难奋进中赢得发展主动权。

做好今年的政府工作，要深入贯彻落实党的十八大和十八届二中、三中全会精神，继续坚持稳中求进工作总基调，努力保持经济持续健康发展和社会和谐稳定。在新的一年里，我们将更加重视增进民生福祉，坚持尽力而为、量力而行，集中更多的财力、精力用于改善和保障民生。我们将更加积极推动改革创新，坚持问题导向，创新发展理念、体制机制和工作措施，为徐州老工业基地全面振兴注入强大动力和活力。我们将更加主动调结构转方式，顺应宏观形势合理安排各项指标，不片面追求发展速度，以科学的考核机制和政策激励机制为导向，把工作重心转向提高发展质量和效益上来。我们将更加有力推进新型城镇化，坚持城乡布局统筹规划、重大项目统筹推进、建设资金统筹安排，全面提升中心城市、中等城市、中小城镇和新农村建设水平，着力破解二元结构，加快缩小城乡差距，把徐州建设成为苏北城乡一体化发展先行区。我们将更加突出生态立市，坚持一手抓污染治理、一手抓生态建设，切实做到多还旧“账”、不欠新“账”，努力让人民群众呼吸清新空气、常享蓝天白云，使良好生态成为徐州新的名片。

到今年底，力争实现以下经济社会发展目标：（1）地区生产总值增长11%左右；（2）公共财政预算收入增长12%；（3）固定资产投资增长19%左右；（4）社会消费品零售总额增长13%左右；（5）实际到帐注册外资增长5%；（6）进出口总额增长5%；（7）城镇居民人均可支配收入和农民人均纯收入增长11%左右；（8）居民消费价格涨幅不高于全省平均水平；（9）科技研发投入占地区生产总值比重达1.9%；（10）城镇登记失业率控制在4%以内；（11）节能减排完成省定任务；（12）人口自然增长率控制在6‰以内。工作中，我们将对照目标要求，细化分解任务，切实抓好落实，务求取得实效，向全市人民交上一份满意的答卷！

三、全面推进各项改革

创造性地落实中央和省关于全面深化改革的决策部署，立足徐州实际大胆实践探索，积极破解制约发展的体制机制约束，以改革的实际成效取信于民。

（一）围绕加快政府职能转变，扎实推进行政体制改革，努力建设效率徐州。全面排查政府管理的“越位点”和“缺位点”，强化政府公共服务、市场监管、环境保护等职能，最大限度减少政府对微观事务的管理。加大行政审批事项“减、转、放、免”力度，力争将行政审批项目全部纳入行政审批服务中心办理。大力推行一次告知、并联审批、限时办结、网上公开办理，加快构建统一规范的政务服务管理平台。建立规范化的政府购买服务机制，凡属事务性管理服务全部纳入购买范围，推广电子订单式政府采购。整合卫生、人口和计生部门的机构和职责，加快地方食品药品监督管理体制改革，年内基本完成市县政府机构改革。开展事业单位分类改革，严格控制人员编制，确保机关事业单位机构编制和财政供养人员总量只减不增。深化投资体制改革，科学界定政府投资范围，健全政府投资项目决策机制。推进财税体制改革，抓好政府收支分类改革，制定实施政府债务举借审核审批工作规程，完善政府债务统计报告制度，逐步将政府债务纳入财政预算管理，建立健全支出绩效评价体系，完善市、区两级政府事权和支出责任更加适应的财政体制，创新财政资金对企业扶持方式。

（二）发挥市场在资源配置中的决定性作用，扎实推进经济体制改革，努力建设活力徐州。实行统一的市场准入制度，在负面清单基础上，促进各类市场主体依法平等进入清单之外的领域，鼓励社会资本投向社会事业、基础设施建设等，清理和废除阻碍公平竞争的障碍，营造更加良好的市场环境。支持非公资本参与国有企业改制重组与合资经营，积极发展混合所有制经济。分类实施国有企业改革，对资产规模较小的企业实施产权制度改革；探索组建管理公司，受政府委托管理国有投融资业务；对资产规模大、改革难度大的国有企业实行管理机制、投资机制改革，探索组建工程机械产业投资基金，构建企业管理团队利益与企业发展绩效密切联系的激励约束机制。健全国有资本经营预算制度，将国有企业全部纳入统一监管和考核体系。加快公共资源交易中心建设。稳妥推进不动产统一登记，实施城镇房屋产权证和土地使用证“两证合一”。统筹抓好农村各项改革。

（三）立足更好保障和改善民生，扎实推进社会体制改革，努力建设幸福徐州。加快教育领域综合改革，推动主城区完全中学初高中分离，实施集团化办学试点，健全中职、高职、本科有机衔接的培养机制。抓好市级公立医院改革试点，深化县级公立医院综合改革，鼓励社会办医，扩大医疗行业对境内外资本开放。完善市级创业服务平台，构建政策扶持、创业培训、创业服务三位一体的工作机制，加快创建省级创业型城市。推行城镇职工补充医疗保险和居民大病保险，完善异地就医管理办法。抓好新农合混合支付方式改革，委托具有资质的商业保险机构开展新农合经办服务。推进退役士兵安置、优待抚恤、最低生活保障、特殊困难残疾人救助等城乡统一标准。创新城乡社区综合服务中心、居家养老服务站运营管理机制，支持多元投资兴办养老机构。改进社会治理方式，深化街道居委会自治试点，激发社会组织活力。健全重大决策社会稳定风险评估机制，实行重大敏感事项“第三方”稳评制度。

（四）推动文化大发展大繁荣，扎实推进文化体制改革，努力建设文化徐州。完善徐州报业传媒、广电传媒、演艺、文产“四大集团”法人治理结构，加快县级经营性文化事业单位、国有文艺院团转企改制和文化市场综合执法机构改革，抓好市级非时政类报刊出版单位转企改制，深化公益性文化单位内部机制改革。实施“舞动汉风”工程，健全舞台艺术生产、文艺精品评价激励等机制，发挥“汉风文华奖”激励导向作用，打响“书画徐州”品牌。鼓励社会力量参与文化建设，采取政府购买、项目补贴、委托生产等多种形式，引导支持民间力量生产公共文化产品、举办公益性文化活动、投资建设文化设施。筹划设立文化产业创业投资基金，大力发展民办民营文化企业，继续推进淮海文博园、动漫文化产业园等园区建设，积极争创省级文化产业示范园区，加快发展壮大文化产业。

（五）更加注重人与自然和谐共生，扎实推进生态文明体制改革，努力建设美丽徐州。建立健全“五大行动计划”组织协调、工作推进、督查考核机制。实施主体功能区规划，积极推进国土空间规划，从严控制土地开发强度，健全城市绿线制度，切实守住生态红线。加快自然资源及其产品价格改革，加大差别化资源价格和惩罚性价格实施力度，探索建立污染物排放指标有偿分配机制和排污权交易机制，开展排污权交易试点，积极推进环境监测、评价等环保服务外包。建立生态文明建设基金，组建“守望家园”志愿者队伍，引导社会资本投入生态建设和环境保护。启动建设骆马湖第二水源地，强化饮用水源地保护，健全城乡统筹区域供水体制，建立污水治理和监管机制。完善国有林区经营管理体制，切实保护林业生态。

四、加快推动经济社会转型发展

在保持经济平稳健康增长的同时，把加快转型发展作为刻不容缓的重大任务，大力推进经济转型、城市转型、生态转型和社会转型。

（一）突出民生优先、和谐共建，在民生改善和社会治理上争取更大成效。深入实施民生幸福工程，着力构建六大公共服务体系，办好11大类100件民生实事，促进人民幸福指数与经济发展水平同步提升。

拓展居民增收和就业创业渠道。突出农民、企业职工、中低收入者和困难家庭等重点群体，健全完善就业创业、社会保障和扶贫开发机制，促进居民收入普遍较快增长。重点落实好黄墩湖滞洪区和黄河故道沿线扶贫开发项目，发放扶贫小额贷款12亿元以上，年内力争实现27万农村低收入人口脱贫。抓好劳动技能培训和新成长劳动力技能培训，提升就业创业能力，推动实现更高质量的就业，力争城镇新增就业10万人、城镇失业人员再就业4万人、农村劳动力转移5万人。以促进大学生创业激发全民创业热情，新增大学生创业企业1200家、带动就业6000人以上。

健全社会保障和住房保障体系。大力推进社会保险扩面征缴工作，实现“五险一票征收”，适时提高企业退休人员基本养老金、城乡居民养老保险基础养老金、城乡低保、新农合保障水平，发行190万张加载金融功能的社会保障卡。完善社会救助制度，新建15个城市小型托老所、25个农村老年关爱之家、100个妇女儿童之家，为主城区10万名60周岁以上老人和残疾人免费办理意外伤害保险。年内建成保障性住房7000套，启动实施市区1000万平方米棚户区、城中村改造和800万平方米安置房建设，改造22个老旧小区，切实改善市民居住条件。

加快发展各项社会事业。推进省学前教育改革发展示范区、义务教育优质均衡发展示范区、职业教育创新发展实验区“三区同建”，新建改扩建中小学38所、幼儿园80所，加固重建中小学校舍60万平方米，加快新一轮教育现代化建设步伐。抓好北区股份制医院、徐医附院开发区分院等4家综合性医院建设，推进县镇村卫生机构标准化建设，争创国家卫生城市。完善公共文化服务体系，建成市县镇村四级公共文化设施网络，继续实施文化下乡等惠民项目，加强文化遗产保护、传承与利用，争创省公共文化服务体系示范区。精心做好第十八届省运会的筹备工作，努力办出一届精彩圆满、节俭创新的省运会；实施“五大健身计划”，广泛开展“全民健身年”活动，推动群众体育和竞技体育全面发展。继续稳定低生育水平，优化出生人口性别比，适时启动实施“单独二孩”政策。抓好全国文明城市创建，做好科普工作，不断提高居民素养。统筹抓好其他各项事业，推动社会全面进步。

促进社会更加和谐稳定。深入实施“三建设三提升”工程，全面深化平安徐州和法治徐州建设。健全立体化治安防控体系，加强城区警务服务站和农村中心警务室建设，大力预防和依法惩治违法犯罪。深入开展信访积案集中化解“百日百案”活动，推动领导干部接访下访常态化，实行涉法涉诉信访依法终结，坚决防止群体性事件和个人极端事件发生。创新社会矛盾纠纷排查调处工作专业化、职业化机制，创建“12348”公共服务平台，打造“多调对接”的人民调解徐州模式。加快产品质量检验检测平台建设，深入开展质量强市活动。严格落实安全生产责任，健全隐患排查治理和预防控制体系，抓好食品药品、安全生产专项整治，强化应急管理，坚决遏制重特大安全事故发生。

（二）突出转型升级、提质增效，在经济结构调整上实现更大突破。瞄准构建现代产业体系这一主攻方向，紧抓增强自主创新能力这一核心环节，加快经济结构战略性调整，打造徐州经济“升级版”。

不断增强经济发展动力。坚持把项目建设作为增后劲调结构的有力抓手，计划安排重大项目200项、总投资3623亿元，其中当年实施180项、年度投资983.5亿元。加快实施郑徐客专、轨道交通1号线一期、观音机场二期等重大基础设施项目，加大徐工重型汽车、升华电梯制造、中能技改、雨润物流中心、龙英纺织等重大产业项目推进力度，力争徐宿淮盐铁路等20项前期推进项目取得实质性进展。坚持“四招四引”，突出抓好产业链招商、专题招商和以商引商，创新招商活动形式，精心组织好北京、上海、台湾、香港、新加坡、日本等系列招商活动。着力稳定外贸出口，支持企业加大国际市场开拓力度，积极推进保税物流中心建设、申报国家级工程机械和地坪地材出口基地。坚持提升硬件和优化环境双管齐下，加快中心商圈、新城区商圈、西部商圈、高铁生态商务区等重点商贸板块建设，提升特色商业街区服务功能，积极促进信息消费，从严打击价格欺诈、假冒伪劣等行为，营造安全放心的消费环境，打造区域消费高地。

加大产业结构调整力度。立足做大做强新兴产业，深入实施培育和发展战略性新兴产业三年行动计划，抓住项目建设、培育领军企业、突破关键技术、创建特色基地等关键环节，推动新能源、高端装备制造和新材料等九大新兴产业发展壮大，加快将徐州打造成为具国际竞争力的太阳能光伏产业基地、高端装备研发和制造基地；引导各县（市）发挥自身优势，重点培育2-3个新兴产业。立足改造提升传统优势产业，拉长增粗六大千亿元工业产业链条，鼓励企业广泛运用信息技术改造提升传统的技术、工艺、设备和营销管理方式，争创国家级、省级两化融合试点（示范）企业，提高产品技术含量和附加值；对接国家和省化解产能过剩方案，坚决淘汰落后产能。立足加快发展现代服务业，突出抓好必康物流分拨中心、综合物流园、钢铁再生利用交易中心等重点项目，健全物流配送体系，推动商贸物流业扩容提质；抓好云龙湖5A级景区创建，规划建设贾汪旅游集聚区，加快旅游公共服务体系建设，打造知名旅游目的地城市；创新服务理念、商业模式和技术手段，大力培育电子商务、文化创意、总部经济、现代金融等服务业态，积极发展物流快递、工业设计等生产性服务业及家政、养老、健康等生活性服务业，促进服务业发展提速、比重提高、结构提升。

大力扶持实体经济。坚持扶大扶优扶强，建立重点企业联系制度和百亿规模企业服务绿色通道，鼓励有条件的企业实施兼并重组、向上下游辐射延伸，打造一批主业突出、带动能力强的龙头企业。对100家成长型中小企业实施对口帮扶，在融资担保、企业上市、教育培训等方面提供全方位服务，引导企业建立现代管理制度，扶持中小企业加速成长。积极开展为企业“送政策、送服务”活动，全面落实“营改增”等财税扶持优惠政策，进一步做好银企对接，及时帮助企业解决生产经营难题。规范实施增减挂钩和土地综合整治工程试点，继续加大工矿废弃地复垦利用力度，保障项目建设和企业发展用地需求。

提高集约集聚发展水平。强化开发园区在经济发展中的主阵地作用，加大简政放权力度，健全开发区考核机制，提升园区综合承载力和集聚吸纳力，促进主导和新兴产业向园区集中集聚。支持徐州经济技术开发区创建国家级生态工业园区，打造太阳能光伏、高端装备制造、生物医药、电子信息等产业链。推动高新区创建科技部创新型产业集群试点，扶持泉山开发区完善配套基础设施，帮助新沂开发区申报国家级开发区，指导睢宁临空产业园、铜山利国工业园、贾汪江庄工业园申报省级开发区，支持丰县、沛县、邳州开发区创建创新型园区。坚持优进劣出、动态发展，推进50家省市级现代服务业集聚区提档升级，加快建设一批农业产业园区和农产品加工集中区。

积极构建区域创新体系。坚持科教与人才强市战略，统筹抓好各类人才队伍建设，重点引进和培养高层次创新创业人才和领军团队。突出企业技术创新主体地位，支持企业通过合作研发、人才引进、参股并购等方式开展创新合作，确保新增省级高新技术企业30家以上。加快科技创新载体建设，力争新建省级以上研发机构20家，帮助江苏师大科技园创建国家级大学科技园、徐州工程学院科技园创建省级大学科技园。深化产学研合作，构建多元化科技投融资体系，鼓励高校院所在徐设立技术转移中心，新建校企联盟180个以上。以创建国家知识产权示范市为契机，强化科技成果转化、名牌产品创建、专利申请和保护，力争企业专利申请量和授权量实现较快增长。

（三）突出城乡一体、产城融合，在推进新型城镇化上迈出更大步伐。着力在优化布局、完善功能和统筹发展上下功夫，加快构建“1530”新型城镇体系，夯实城镇化的产业支撑，形成城乡一体、共同繁荣的新格局。

高水平建设区域中心城市。围绕构建“八大中心”，进一步完善城市规划体系，实施八大类258项城建重点工程。在继续推进老城区改造的同时，抓好新城区沿昆仑大道、汉源大道、迎宾大道和环大龙湖“四轴”开发，促进徐州经济技术开发区、铜山区、贾汪区与主城区有机融合。着眼构建立体交通体系，重点推进三环西路北路高架、徐沛快速路、外环公路一期等道路畅通工程，继续整修城区道路，大力发展城市公共交通，切实规范交通秩序，最大限度缓解交通拥堵。抓好中心商圈、龟山小镇、万科新天地、铜山万达广场等商贸旅游项目和奥体中心、市民文化活动中心等文化体育项目建设，大力发展城市经济，促进城市功能形态双提升。加强“智慧城市”建设，积极推进部省市共建主城区城市地质工程。完善数字化城管平台，进一步理顺城管执法体制，力争跻身首批“江苏省优秀管理城市”；继续开展“外创示范道路、内创幸福家园”活动，强化背街小巷、老旧小区环境卫生专项整治，积极创建省级城市管理示范社区；实施环卫保洁第三方监理机制，推行垃圾收集转运和路灯、道路、桥梁维护管养的市场化运作。

进一步完善新型城镇体系。注重以城带乡、产城融合，做好县（市）总体规划修编，促进城镇规划、土地利用规划、产业发展规划、生态建设规划等有机衔接，建立“一张图”综合信息平台。加强5个县级中等城市建设，促进老城区与新城区、开发区融合发展，提升人口吸纳力和产业承载力。抓好第三批中心镇达标验收命名和重点中心镇提档升级，深入实施管理示范镇创建，推进扩权强镇改革试点，打造特色新型城镇群。放开建制镇和小城市落户限制，推进农业转移人口有序转为城镇居民。新建50个新农村示范村。建成农村公路400公里，改造危桥119座，完成改厕6万户，推动交通、电信、卫生、文化、邮政快递等基础设施向农村延伸。实施“以市带县（区）融资模式”，支持县（区）棚户区改造。

加快推进县域经济发展壮大。坚持新型工业化、新型城镇化和农业现代化互动融合，鼓励各地因地制宜打造特色产业、加快城镇化步伐，力争年内县域城镇化水平提高2个百分点以上。扎实推进新一轮“丰县崛起”行动计划，赋予“突破睢宁”战略新内涵，确保丰县、睢宁达到省全面小康评定标准；加快促进沛北一体化，支持邳州、新沂创建国家环保模范城市；加强铜山区基础设施、公共服务与主城区的对接，加快贾汪区资源枯竭城市转型发展，促进县域争先进位，增强发展活力和整体实力。

加速推进农业现代化发展。稳定粮食生产，确保粮食安全。探索新型农业生产经营模式，积极稳妥推进土地流转，新增农田托管65万亩以上，壮大农民专业合作和家庭农场，发展多种形式的适度规模经营。继续实施“2020工程”，新增和改造升级设施农业20万亩，新建扩建大中型畜禽规模养殖场20个。深入推进黄河故道综合开发，切实加强9个农业示范区、吕梁风景区10个生态农场建设。加快农业产业化步伐，抓好农业龙头企业培育、农产品流通体系建设、农业标准化生产、农技推广服务体系完善等环节，发展壮大果品、肉鸭、奶业、大蒜和食用菌5大主导产业。加强农田水利建设和水利工程管理，继续实施山区水源工程，加快水利现代化建设步伐。抓好中低产田改造和粮食高产创建，提高农业机械化水平和综合生产能力。

（四）突出绿色发展、优美宜居，在生态文明建设上力争更大作为。注重开发和保护并重、污染防治和生态修复并举，标本兼治，综合施策，深入实施“五大行动计划”，确保天更蓝、水更清、地更绿、路更畅、城更靓。

一着不让抓好节能减排。完善节能预警调控机制，加快节能技术、产品的开发和推广应用，切实抓好工业、建筑、交通、公共机构和居民生活等领域的节能工作，有序推进281个治污项目，确保完成省下达的节能减排任务。把住源头严控增量，严格执行规划及新建项目能耗和环境影响评价制度，坚决杜绝高能耗、高污染、高排放、低效益项目上马，不再新上单纯扩大产能的传统产业项目。多策并举消化存量，组织开展重点企业节能低碳行动和能效水平对标达标活动，推广合同能源管理、余热余压利用等节能措施，力争全面完成年耗能3000吨标准煤以上企业的节能改造。

切实解决重点环境问题。完善大气污染联防联控联治

体系，加大老旧机动车淘汰报废力度，加强秸秆综合利用和禁烧，综合治理工业废气、煤烟污染、建筑扬尘和露天烧烤，全部关闭城区小热电企业，加快推进企业退城入园，切实减少灰霾污染，提升城区环境空气质量。抓好污水处理厂及管网建设和运营监管，确保现有污水处理厂运行负荷达到80%以上，在建的镇级污水处理厂及配套管网建设覆盖率达到85%。对故黄河、三八河等19条骨干河道实施截污清淤贯通，基本消除城市黑臭河流；深化调水通道82条主支河流的环境综合整治，完成丰县、沛县、睢宁尾水资源化利用工程，保证南水北调断面水质稳定达标。

加强生态保护和生态创建。落实最严格的环保准入门槛、污染排放标准和环保问责制度，大力促进绿色低碳发展。绿化荒山1.7万亩，全面完成“二次进军荒山”计划。开展泉山森林公园、汉文化景区敞园改造，精心打造奥体公园、吕梁风景区、潘安湖湿地公园、丁万河景观带，抓好三环东路、四环高速、4万亩黄河故道沿线绿化带等绿化项目建设，大规模构建城乡绿色屏障。实施街头绿地、滨河公园建设和道路景观绿化工程，对湖东路、迎宾路等道路进行绿化提升改造，继续抓好采石宕口、采煤塌陷地生态修复，确保市区建成区道路绿化普及率达到100%、公园绿地500米服务半径覆盖率达到90%。深入实施美好城乡建设行动，分类推进村镇环境整治，完善垃圾收运、污水处理等环卫设施，实现城乡面貌更大改观。对照生态创建整体进度，抓好对标找差，力争尽快达到国家生态市和生态园林城市创建标准。

五、努力建设法治政府和服务型政府

做好今年经济社会发展各项工作，必须进一步加强政府自身建设，切实促进政府职能转变、规范行政权力运行、提高政府效率和效能。

（一）强化公开透明，大力推进行政权力阳光运行。深化和扩大政务公开，提升行政权力网上公开透明运行水平，努力实现权力运行规则公开、程序公开、过程公开、结果公开，以公开提高政府公信力。不断扩大财政预决算公开范围，年内市直所有预算单位全面推行部门预决算公开，逐步推进行政经费决算公开、三公经费公开和重大财政专项支出公开。拓展和完善“徐州发布”平台，及时高效做好信息发布工作，畅通政府与公众交流互动渠道。

（二）坚持依法合规，全面提升行政行为规范化水平。健全决策、执行、监督相互制约又相互协调的运行机制，确保政府行为于法有据、程序正当。完善行政决策机制，把公众参与、专家论证、风险评估、合法性审查、集体讨论决定等作为重大决策必经程序，提升行政决策的科学化水平。强化立法前期论证，积极开展立法后评估工作，促进“立、改、废”有机结合，提高政府立法科学性。加强行政执法和行政复议规范化建设，进一步规范行政执法自由裁量权。自觉接受市人大及其常委会的法律监督和工作监督，主动接受市政协的民主监督，重视社会公众监督和舆论监督，加强行政监察和审计监督。

（三）立足高效便捷，进一步提高行政服务效率效能。推进百姓办事“零障碍”工程全覆盖，简化行政流程，认真落实服务承诺、首问负责和限时办结等制度，探索构建标准化服务体系。抓好“12345”政府服务热线运行管理，办好行风热线和“直通12345”电视专栏，打造“线报声屏网”立体联动体系。增强行政服务中心、便民服务中心功能，探索建立网上办事大厅。加快电子政务建设，完善政府门户网站功能，推进政府系统办公自动化。加强行政问责和政务督查，提高政府执行力。抓好第三次全国经济普查工作，健全统计监测评价体系，为决策提供科学依据。

（四）突出务实清廉，切实营造风清气正的政务环境。扎实开展群众路线教育实践活动，严格落实关于改进工作作风、密切联系群众的各项规定，着力解决群众反映强烈的突出问题。严格执行《党政机关厉行节约反对浪费条例》，控制和压缩“三公”经费和一般性支出，从源头上狠刹奢侈浪费之风。规范创建达标检查评比表彰和庆典、论坛、展会等活动，规范配置办公用房，规范公务接待，继续精简会议和文件，切实改进会风文风，用更多的时间调查研究、深入基层。完善惩治和预防腐败体系，加强政风行风建设，深入开展“两个习惯”教育，坚决查处领导干部违法违纪案件，努力建设人民更加信赖的政府。

各位代表，徐州正处于跨越发展、转型发展的关键时期，我们肩负的任务艰巨而繁重、责任重大而光荣。让我们紧密团结在以习近平同志为总书记的党中央周围，全面贯彻落实省委、省政府和市委的决策部署，群策群力，开拓创新，扎实工作，为在新的起点上开创徐州“两个率先”新局面而努力奋斗！
